# Self-administration of adrenaline for anaphylaxis during in-hospital food challenges improves health-related quality of life

**DOI:** 10.1136/archdischild-2020-319906

**Published:** 2020-09-18

**Authors:** Sarah Burrell, Nandinee Patel, Marta Vazquez-Ortiz, Dianne E. Campbell, Audrey DunnGalvin, Paul J. Turner

**Affiliations:** 1 Section of Inflammation, Repair and Development, National Heart & Lung Institute, Imperial College London, London, UK; 2 Discipline of Child and Adolescent Health, University of Sydney, Sydney, New South Wales, Australia; 3 School of Applied Psychology, Department of Paediatrics and Child Health, University College Cork, Cork, Ireland

**Keywords:** adolescent health, therapeutics

## Abstract

**Objective:**

To assess the impact of anaphylaxis on health-related quality of life (HRQL) and self-efficacy in food-allergic patients undergoing in-hospital food challenge.

**Design:**

Secondary analysis of a randomised controlled trial.

**Setting:**

Specialist allergy centre.

**Patients:**

Peanut-allergic young people aged 8–16 years.

**Interventions:**

Double-blind, placebo-controlled food challenge to peanut, with HRQL and self-efficacy assessed using validated questionnaire, approximately 2 weeks prior to and 2 weeks after challenge. Where possible, anaphylaxis was treated with self-injected adrenaline (epinephrine).

**Main outcome measures:**

Change in HRQL and self-efficacy.

**Results:**

56 participants had reactions at food challenge, of whom 16 (29%) had anaphylaxis. Overall, there was an improvement in HRQL (mean 2.6 points (95% CI 0.3 to 4.8); p=0.030) and self-efficacy (mean 4.1 points (95% CI 2.4 to 5.9); p<0.0001), independent of whether anaphylaxis occurred. Parents also reported improved HRQL (mean 10.3 points (95% CI 5.9 to 14.7); p<0.0001). We found evidence of discordance between the improvement in HRQL and self-efficacy as reported by young people and that perceived by parents in their child.

**Conclusions:**

Anaphylaxis at food challenge, followed by self-administration of injected adrenaline, was associated with an increase in HRQL and self-efficacy in young people with peanut allergy. We found no evidence that the occurrence of anaphylaxis had a detrimental effect. Young people should be encouraged to self-administer adrenaline using their autoinjector device to treat anaphylaxis at in-hospital challenge.

**Trial registration number:**

NCT02149719

**What is already known on this topic?**

- Suspicion of food allergy is often confirmed at in-hospital, supervised food challenge.
- Food challenges have a positive impact on health-related quality of life, even when this causes a clinical reaction; this has not been evaluated for anaphylaxis.
- Anaphylaxis is common but is typically undertreated, even at in-hospital supervised food challenge.

**What this study adds?**

- The improvement in health-related quality of life and self-efficacy following food challenge occurs, even when this caused anaphylaxis.
- The occurrence of anaphylaxis provides an opportunity for patients to self-inject with adrenaline and results in a significant improvement in self-efficacy.
- There is significant discordance between health-related quality of life reported by young people with peanut allergy and their parents: both need to be assessed independently.

## Introduction

Food allergy is recognised as a substantial public health burden in many countries. IgE-mediated food allergy is estimated to affect 2%–3% of children.^1^ It is the most common cause of potentially life-threatening anaphylaxis in this age group.^2^ Between 1998 and 2012, there were a total of 14 675 hospital admissions coded as food-induced anaphylaxis in the England and Wales.^2^ The fear of anaphylaxis and need for dietary limitation results in a significant impairment in health-related quality of life (HRQL) in affected individuals and their families.^3^

Ideally, food allergy (and its persistence or resolution) should be confirmed through controlled exposure to the food at a supervised oral food challenge. However, food challenges are costly, time-consuming and not without risk (with potential for anaphylaxis). In practice, given limited clinical resources, surrogate measures—the detection of allergen-specific IgE (in a skin prick test or in blood)—are used to identify ‘sensitisation’. Unfortunately, sensitisation frequently fails to correlate with true clinical reactivity: typically, low diagnostic cut-offs with a high sensitivity (but low specificity) are used, resulting in a significant false positive rate of over 50% in population-based studies.^4–6^ This causes overdiagnosis, increasing the adverse impact on affected individuals and their families and leads to unnecessary dietary/social restrictions. This in turn drives further demand for food challenges to confirm diagnosis.

Current management according to national and international guidelines recommend intramuscular adrenaline (epinephrine) as first-line treatment for anaphylaxis.^7–10^ Individuals at risk of anaphylaxis should be prescribed adrenaline autoinjectors (AAIs) for self-administration as rescue treatment.^10^ Delays in treatment with adrenaline are associated with fatal outcomes.^11^ Up to 30% of food challenges in specialist centres, and 40% of reactions due to accidental exposure outside hospital, cause anaphylaxis.^12^ However, there is significant underuse of AAI to treat anaphylaxis, both in the prehospital environment and in hospital departments.^8 11–15^ This may relate to a failure to recognise anaphylaxis, or a reluctance to use an AAI, for example, due to fear of injection^16^ or through delaying discharge after a planned food challenge.

Oral immunotherapy is an emerging treatment option for some food allergies^17^; most protocols require participants to undergo an initial food challenge to confirm clinical reactivity. In this context, anaphylaxis may be more common, as participants are more likely to have true underlying food allergy. In addition, such protocols generally mandate the use of more objective criteria to determine a ‘positive’ challenge, which might increase the likelihood of anaphylaxis compared with more routine challenges where often softer symptoms result in earlier stopping and thus a lower cumulative allergen exposure. Anaphylaxis at food challenge could have a negative impact on the young person in terms of HRQL or that of their parent/carer. In addition, it may affect the young person’s perceived ability and knowledge to self-manage an anaphylaxis reaction (a concept described as self-efficacy). We therefore sought to assess the impact of anaphylaxis on HRQL and self-efficacy in patients undergoing food challenge prior to commencing oral immunotherapy.

## Methods

### Study design

This is a secondary analysis of children undergoing double-blind, placebo-controlled food challenge (DBPCFC) to peanut as part of screening procedures for a single-centre, open-label, randomised study examining oral peanut immunotherapy (BOPI Study, Clinical Trials.gov NCT02149719.). The study was overseen by an independent safety and data monitoring committee.

### Participants

Young people (age 8–16 years) with a diagnosis of peanut allergy were recruited from local allergy clinics, and nationally (through the Anaphylaxis Campaign patient support group), either through referral from their healthcare provider or by self-referral. Informed written consent was obtained from the parent/guardian, together with written assent from the young person. This age group was chosen as we considered age 8 years to be the youngest age at which young people can reliably demonstrate the necessary comprehension to give valid consent/assent for oral immunotherapy studies. In addition, the questionnaires used to assess HRQL have only been validated in children age 8+ years.^3 18^ Participants were excluded if they had any of the following: a clinically significant chronic illness (other than asthma, eczema or allergic rhinitis); current use of anti-IgE therapy; on immunosuppression, beta-blockers or ACE-inhibitors; poorly controlled asthma within the previous 3 months; or a previous intensive care admission for management of anaphylaxis. Participants with a prior history of anaphylaxis (not requiring intensive care) were not excluded.

### Procedures

Skin prick testing (SPT) was performed to peanut extract (ALK-Abello, Hørsholm, Denmark) using single-point lancets, according to national guidelines. Histamine (10 mg/mL, Stallergenes, UK) was used as a positive control. A positive SPT was defined as a weal size of at least 3 mm greater than a negative control read at 15 min. Total and peanut-specific IgE was measured with the ImmunoCap system (Thermo Fisher, Uppsala, Sweden).

DBPCFC to peanut were conducted according to international PRACTALL consensus criteria.^19^ Prior to challenge, participants had tolerance confirmed to the FC matrix (WOW butter, Hilton Whole Grain Millers Ltd, Canada), which is soya based. Challenge doses were administered as a ‘mini-sandwich’ consisting of bread with the WOW butter inside; active doses also included the appropriate dose of defatted roasted peanut flour (Golden Peanut Company, Albany, Georgia; 12% fat). The two visits (active and placebo) were conducted on separate occasions, at least 14 days apart. On each day, subjects received incremental doses, every 30 min, of peanut protein (or placebo) at the following doses: 3 mg, 10 mg, 30 mg, 100 mg, 300 mg, 1000 mg and 3000 mg until stopping criteria were met.^19^ The order of visits was determined by a computer-generated randomisation table. Members of the research team were blinded as to the challenge assignment, aside from the technician preparing the challenge material. Anaphylaxis was defined according to UK definitions to include only those patients with objective respiratory or cardiovascular signs^8^; where possible, this was treated through self-administration of adrenaline by participants using their AAI.

HRQL and self-efficacy assessments were undertaken at least 2 weeks prior to challenge, and approximately 2 weeks following completion of both challenge visits, using previously validated questionnaires, in both parents and young people independently.^18 20 21^ The Food Allergy Quality of Life Questionnaire (FAQLQ-10) measures disease-specific HRQL, that is, the impact of food allergy on HRQL in the young person, as assessed by themselves (FAQLQ-10-CF or FAQLQ-10-TF) or as perceived by the parent in their child (parent proxy, FAQLQ-10-PF).^18^ The impact of the child’s diagnosis on the parent themselves was assessed by FAQLQ-Parent Burden (FAQLQ-PB).^20^ Questions were scored on a Likert scale of 0–6, with a minimal clinically important difference of 0.5 points; higher scores are associated with a greater adverse impact on HRQL. Self-efficacy measures the individual’s confidence and ability to manage their food allergy (or in the case of the questionnaire completed by the parents, the parent’s perception of their child’s confidence to manage their allergy).^21^ The lower the score, the greater the negative impact on the individual, meaning the individual is less confident.

### Statistical analysis

Normality of data was assessed using Shapiro-Wilk test, and data were subsequently analysed using the appropriate parametric (t-test) or non-parametric test (Mann-Whitney test for unpaired comparisons and Wilcoxon signed-rank for paired data) (Prism 8, GraphPad Software, California, USA). A study sample size of at least 46 peanut-allergic children was determined for the intended primary outcome measure of the BOPI Study. Recruitment continued until this sample size was achieved, resulting in 68 participants undergoing study screening including DBPCFC.

## Results

### Study population/patient characteristics

Eighty-five young people were screened for the study, of whom 68 underwent food challenge between July 2015 and January 2017; 56 reacted at food challenge and returned for follow-up (see Consolidated Standards of Reporting Trials diagram, online supplemental figure S1). Baseline characteristics are shown in table 1. The symptoms experienced at challenge are summarised in table 2; 16 (29%) participants had anaphylaxis, of whom 14 self-administered adrenaline using their AAI (in the remaining two cases, the AAI was administered by the parent). There were no significant differences in baseline characteristics between those who experienced anaphylaxis at challenge and those who did not, with the exception that the former were more likely to have a history of prior anaphylaxis to peanut (p<0.0001, Fisher’s exact test).

10.1136/archdischild-2020-319906.supp1 Supplementary data

**Table 1:** Baseline descriptive characteristics of participants

	Overall cohort n=56	Mild reaction n=40	Anaphylaxis n=16
Age (years)	12.7 (8–16.9)	12.4 (8–16.9)	13.3 (8.5–15.6)
Gender (% male)	55	52	62
Age at diagnosis (years)	2.5 (1–14)	2.5 (1–12)	2.5 (1–14)
Previous anaphylaxis to peanut, n (%)	23 (41)	11 (27)	12 (75)
Concomitant atopic disease, n (%):			
Allergy to tree nuts	10 (18)	9 (22)	1 (6)
Asthma	39 (70)	26 (65)	13 (81)
Allergic rhinitis	44 (79)	33 (82)	11 (69)
Eczema	30 (54)	22 (55)	8 (50)
Skin prick test to peanut (mm)	9 (3–22)	9 (3–22)	8 (4–17)
Serum IgE (kUA/L) to:			
Peanut	60.8 (0.6 to >100)	51.4 (1.0 to >100)	77.2 (0.6 to >100)
Ara h 1	6.8 (>0.1 to <100)	4.1 (<0.1 to <100)	31.5 (>0.1 to <100)
Ara h 2	26.2 (0.2 to >100)	26.2 (0.6 to <100)	38.5 (0.21 to <100)
Ara h 3	0.6 (>0.1 to <100)	0.4 (>0.1 to 79.3)	2.57(>0.1 to <100)
Eliciting dose at challenge (mg peanut protein)	143 (3–4443)	43 (3–1443)	143 (13–4443)

Data expressed as median (range) where appropriate.

**Table 2:** Symptoms experienced at food challenge

	Overall cohort (n=56) (%)	Mild (n=40) (%)	Anaphylaxis (n=16) (%)
Skin	96	95	100
Angioedema	78	75	87
Erythema	66	72	50
Urticaria	52	50	56
Upper respiratory	66	57	87
Transient nasal symptoms	11	7	19
Mild rhinitis/eye itch	37	30	56
Severe rhinitis	12	15	6
Laryngeal	93	90	100
Oropharyngeal itch	41	52	12
Throat clearing/throat tightness	37	30	56
Soft stridor, hoarse voice	2	0	6
Lower respiratory	36	12	94
Subjective chest tightness	11	10	12
Cough/wheeze	3	0	12
Hypoxia or cyanosis	2	0	12
Gastrointestinal	78	77	81
Transient symptoms	21	15	37
Single episode of emesis/diarrhoea	37	39	31
Persistent vomiting	18	22	6
Neurological			
Change in behaviour	11	12	6
Significant change in mental status	2	0	6

### Change in HRQL following food challenge

**Figure 1:**
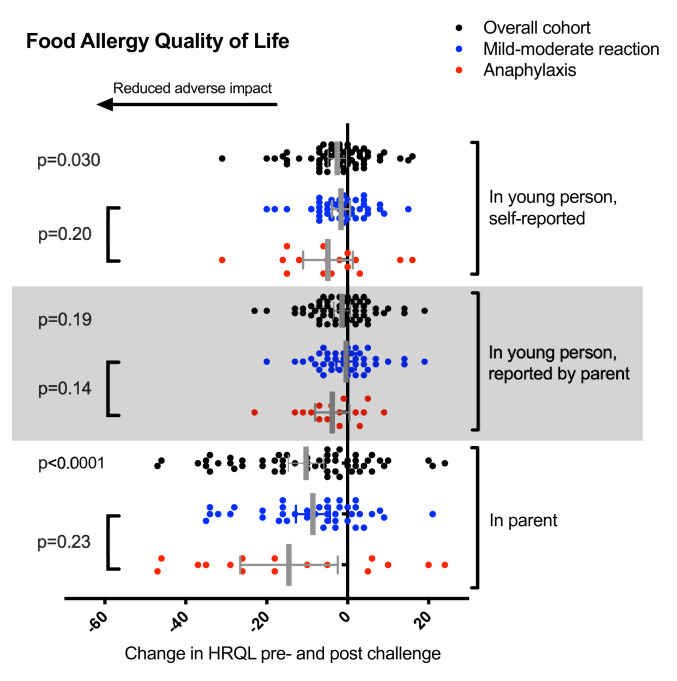
Change in HRQL in the participant (FAQLQ-YP), parent proxy (FAQLQ-P) and the parent themselves following food challenge, overall and by reaction severity. Error bars represent 95% CIs. FAQLQ, Food Allergy Quality of Life Questionnaire; HRQL, health-related quality of life.

Overall, participants reported a statistically significant improvement in FAQLQ (mean improvement 2.6 (95% CI 0.3 to 4.8); p=0.030) (figure 1). This effect was seen, independent of the occurrence of anaphylaxis. We also observed a significant improvement in FAQLQ-PB (mean improvement 10.3 (95% CI 5.9 to 14.7); p<0.0001), that is, the experience of food challenge resulted in a significant improvement in the parent’s own disease-specific anxiety. Interestingly, while there was also a trend in improvement of FAQLQ, as perceived by the parent in their child (mean improvement 1.4 (95% CI −0.7 to 3.4), p=0.19), this did not reach statistical significance.

We evaluated whether the degree of improvement in HRQL with challenge was related to baseline HRQL, as those with a higher baseline FAQLQ (higher disease-related anxiety) might report a greater improvement postchallenge. However, we only found a weak to moderate correlation between baseline HRQL and the improvement postchallenge (Pearson’s R −0.43 for parents, −0.29 for teens and −0.49 for children).

### Self-efficacy

**Figure 2:**
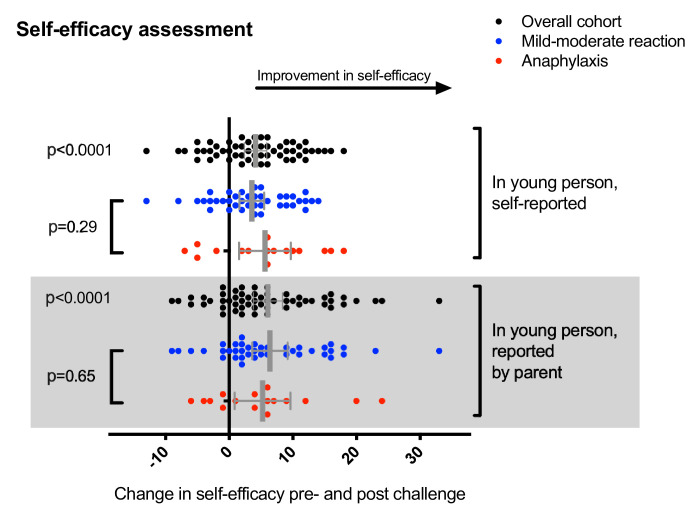
Change in self-efficacy score reported by participants and parent proxy (ie, change in the young person as reported by the parent). Error bars represent 95% CIs.

Study participants reported a significant increase in self-efficacy (mean improvement 4.1 (95% CI 2.4 to 5.9); p<0.0001) and was also noted in the parental assessment of their child’s self-efficacy (mean improvement 6.1 (95% CI 3.8 to 8.3); p<0.0001) following food challenge (figure 2). We observed a trend towards greater improvement in those self-administering adrenaline by AAI, compared with participants with less severe reactions, although this was not statistically significant.

### Discordance in FAQLQ and self-efficacy reporting by parents and their child

To assess for possible concordance in reporting of FAQLQ and self-efficacy by young people and their parent’s perception of the same measure, we evaluated the correlation between the change in HRQL and self-efficacy following challenge as perceived by the parent in their child and that reported by the young person themselves. We found no significant correlation (FAQLQ: r=−0.043, p=0.73; self-efficacy: r=0.10, p=0.45). This implies there was significant discordance between parental perception of the change in their child’s HRQL/self-efficacy following challenge and the same measure reported by the young person themselves.

### Persistence of the improvement in HRQL and self-efficacy

Finally, in 12 participants who did not commence active immunotherapy until 1 year later, we were able to reassess FAQLQ and self-efficacy after 12 months. We did not observe any change in FAQLQ or self-efficacy (online supplemental figure 2).

## Discussion

In this cohort of peanut-allergic young people, experiencing an allergic reaction in hospital under clinical supervision improved HRQL, confirming previous data that did not include any documented cases of anaphylaxis.^22–24^ We further demonstrate that this benefit occurs even after anaphylaxis and is also associated with a significant improvement in self-efficacy.

Self-efficacy describes an individual’s perceived capabilities or knowledge to manage a situation and make changes and captures the individual’s resilience when dealing with the barriers and challenges associated with chronic disease self-management.^25^ It is different to other HRQL measures that look at perception of disease severity and its consequences^26^; nonetheless, the two are codependent: a low level of confidence in managing an allergic reaction will adversely impact on HRQL. In diabetes, self-efficacy has been identified as being particularly important in adolescence, as individuals navigate different developmental stages.^25^ A combination of a good level of knowledge but poor self-efficacy is unlikely to result in improvement alone: studies looking at chronic disease management have shown that a strong self-efficacy is associated with improved management.^27^

Historically, anaphylaxis is undertreated, even when occurring at in-hospital food challenge in specialist centres (table 3),^28–32^ despite this being contradictory to consensus guidelines. This is likely to be due to a combination of reluctance to inject adrenaline by healthcare professionals, patients and parents,^16^ perhaps due to a concern that this may prolong patient discharge. Unfortunately, this reinforces continued poor practice for the non-use of adrenaline to treat anaphylaxis, increasing the risk of adverse outcomes. Our approach encouraged participants to self-administer adrenaline during anaphylaxis; adrenaline autoinjectors caused minimal discomfort when used during this study (see online supplemental video of use of ‘live’ Epipen to treat an episode of anaphylaxis). Fourteen patients were able to do so, and all were discharged after routine monitoring without the need for hospital admission. The experience was associated with an improvement in both HRQL and self-efficacy, on the part of the young person and their parent, although our study was underpowered to assess whether this resulted in greater benefit compared with those who has more mild reactions. Furthermore, the improvements in HRQL and self-efficacy were sustained for at least 1 year, in contrast to previous data demonstrating the loss of effect over time.^23^ Shemesh *et al* 33 reported that in a clinic setting, self-injection with an empty needle and syringe improved a young person’s confidence to use their AAI. To our knowledge, ours is the first systematic analysis of self-administration of AAI during in-hospital anaphylaxis on HRQL and self-efficacy. The ‘mastery experience’ is one of the most powerful tools to increase self-confidence.

10.1136/archdischild-2020-319906.supp2 Supplementary video

**Table 3:** Treatment of anaphylaxis at in-hospital food challenge with adrenaline reported in the literature

Series of in-hospital food challenges reported in the literature	No. of cases of anaphylaxis	Percentage treated with adrenaline
Japan, 2017^28^	190	47
Philadelphia, USA, 2015^29^	655	39
Sydney, 2015^30^	12	42
New York, USA, 2015^31^	42	69
Berlin, Germany, 2014^32^	35	6

In food allergy, self-efficacy has been identified as the main contributing factor impacting on parental anxiety when caring for a food allergic child.^26^ It is therefore interesting that we found significant discordance between HRQL and self-efficacy reported by young people themselves and that reported by their parents using validated parent-proxy questionnaires. In particular, parents appeared to show a greater improvement in their own anxiety (assessed by FAQLQ-PB) than their perceived improvement in HRQL in their child. Our data reinforce the importance of assessing HRQL independently, in both parents and young people.

### Conclusions

In summary, anaphylaxis at food challenge, followed by self-administration of injected adrenaline by young people, was associated with an increase in HRQL and self-efficacy. We found no evidence that the occurrence of anaphylaxis resulted in a detrimental effect. This approach—of self-administration using AAI—should be encouraged by healthcare professionals, both in terms of being consistent with national and international guidelines and to provide invaluable education to patients and their families in the use of AAI.

## Data Availability

Anonymised data are available on reasonable request, from the corresponding author.
