# Supplementary material for: Self-administration of adrenaline for anaphylaxis during in-hospital food challenges improves health-related quality of life
Source: Arch Dis Child. 2020 Sep 18;106(6):558–63. doi: 10.1136/archdischild-2020-319906 (PMC8142442; doi:10.1136/archdischild-2020-319906)

Supplementary Figures

Figure S1: CONSORT diagram

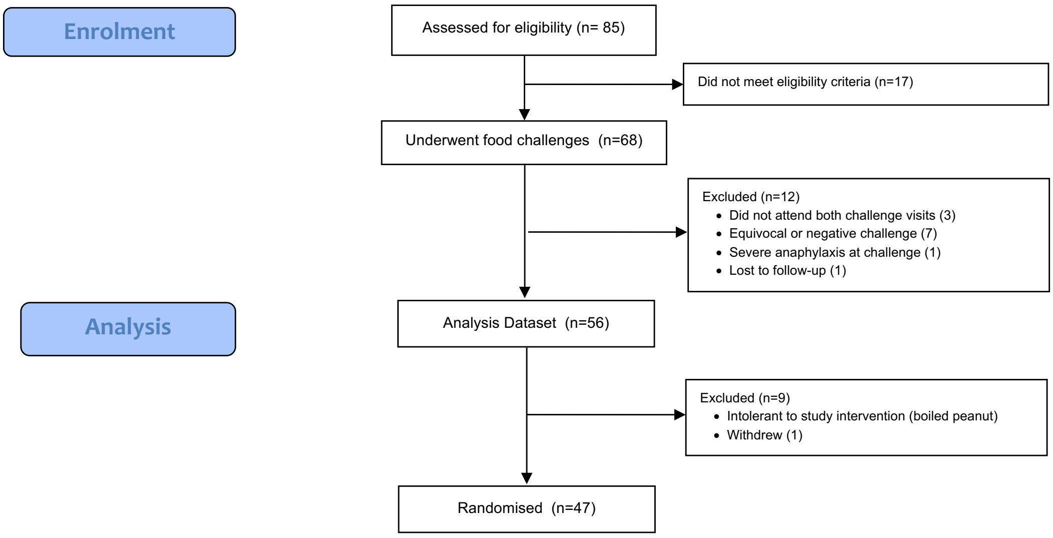

Figure S2: Change in HRQL and self-efficacy score reported by participants their parents, between the challenge and 12 months later.

FAQLQ-YP, HRQL in the young person; FAQLQ-parent, parent proxy report; FAQLQ-PB, HRQL in the parent.

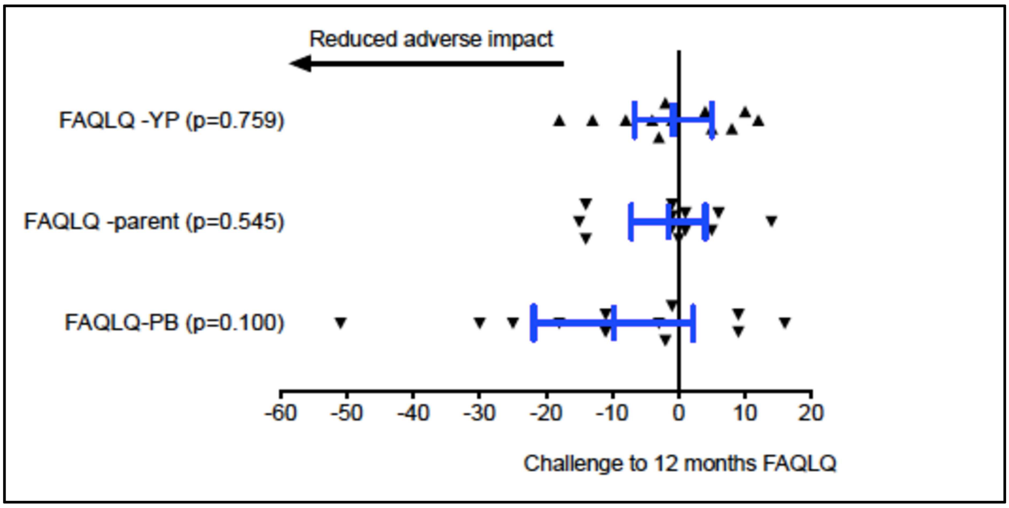

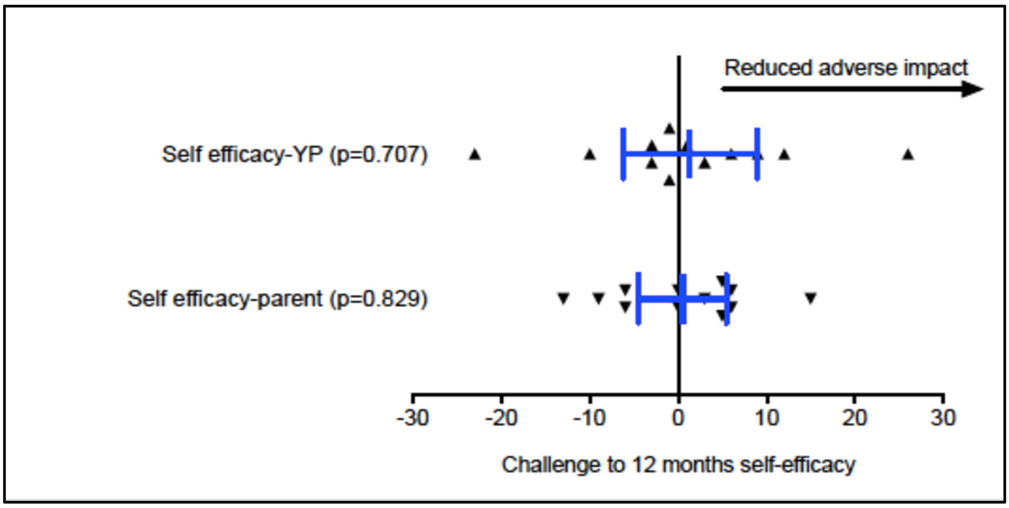

Supplement: Supplementary data [file archdischild-2020-319906supp001.pdf]
